# Practice of pediatric palliative extubation in Brazil: a case series

**DOI:** 10.62675/2965-2774.20250176

**Published:** 2025-04-16

**Authors:** Katarina Maciel Abath, Sheyla Suelle dos Santos Levy, Maria do Carmo Menezes Bezerra Duarte

**Affiliations:** 1 Instituto de Medicina Integral Prof Fernando Figueira Recife PE Brazil Instituto de Medicina Integral Prof. Fernando Figueira - Recife (PE), Brazil.

**Keywords:** Palliative care, Airway extubation, Respiration, artificial, Ventilator weaning, Chronic diseases, Child, Infant, newborn, Patient discharge, Pediatric intensive care units

## Abstract

**Objective:**

To describe the clinical profile, procedures applied and outcomes of patients undergoing palliative extubation in the pediatric intensive care unit at a high-complexity teaching hospital in the northeastern region of Brazil.

**Methods:**

This is a descriptive analysis of a case series that included patients aged under 14 years who underwent palliative extubation in the pediatric intensive care unit between 2016 and 2023 (seven years). Data on admission diagnoses, palliative extubation indications, applied therapies, and outcomes following palliative extubation were retrieved from medical records.

**Results:**

In total, 35 patients were included in the service database. In eight patients, reports could not be found, and these patients were excluded. Twenty-seven patients aged between five days and ten years, mostly females (51.8%) and those with chronic diseases (77.8%), were included in the study. All patients were classified on the basis of World Health Organization pediatric palliative care indication categories. Palliative extubation was considered after the identification of severe neurological impairment, inadequate response or absence of curative therapies, and failure of mechanical ventilation weaning. Palliative care approaches were discussed with the family in 74% of the cases before palliative extubation. Following palliative extubation, 48.1% of patients presented symptoms, and dyspnea (84.6%) and agitation (53.8%) were the most common symptoms. Death occurred in 88.8% of the children from 20 minutes to 38 days after palliative extubation at the hospital. Three children (11.2%) were discharged from the hospital.

**Conclusion:**

Palliative extubation was mostly performed in infants diagnosed with complex chronic conditions and severe and irreversible diseases, all of whom were referred to other palliative care. Death in the hospital while controlling for some symptoms was the main outcome.

## INTRODUCTION

Contemporary scientific and technological development has allowed for the diagnosis and cure of many morbidities and increased the survival of children and adolescents with severe and chronic diseases.^(1)^ Nevertheless, the use of life-sustaining therapies may prolong suffering and death instead of improving quality of life and functionality.^(2)^ A discussion of the limitations of life-sustaining therapies (LLSTs) is associated with the implementation of palliative care practices in pediatric patients.^(2)^ The limitation of life-sustaining therapies refers to do-not-resuscitate (DNR) orders and the withholding, limitation, or withdrawal of life-sustaining therapies, or a combination of these.^(2,3)^ All these decisions are equivalent to relieving suffering and are ethically acceptable.^(2,4)^ However, despite the increasing number of studies on this topic, divergences regarding the definition and practice of LLSTs still exist.^(5)^

Among LLST options, palliative extubation (PE) is the most controversial and challenging decision-making procedure because of the high probability of rapid death.^(4)^ PE is defined as the removal of invasive mechanical ventilation (IMV) when the clinical condition is deemed irreversible and the priority of care is to provide comfort and allow the natural progression of the disease.^(6,7)^ Palliative extubation should be considered when a) IMV weaning fails and its continuation does not align with patient care goals; b) the patient shows significant compromise of quality of life or functionality without clear improvement, highlighting unnecessary suffering from IMV; c) the condition is irreversible and maintaining IMV will only prolong and postpone the death process; d) after successful resuscitation maneuvers, the evaluation shows that the support does not meet the established patient care goals; and e) when the procedure is requested by the patient, if capable of decision-making.^(6)^

Several studies and institutional protocols have been developed to facilitate the decision-making process regarding LLSTs and PE and guide the steps required to ensure its bioethical, legal, and clinical aspects.^(2,7-10)^ However, few studies have focused on the pediatric population, and most of the data are extracted from the adult population. Therefore, this study aimed to describe the clinical characteristics, the procedure of withdrawing IMV, and the outcomes of pediatric patients undergoing PE in two pediatric intensive care units (ICUs).

## METHODS

This study was approved by the ethics committee of *Instituto de Medicina Integral Prof. Fernando Figueira* (IMIP); CAAE: 59743022.8.0000.5201.

This is a descriptive case series study of patients admitted to two pediatric ICUs who underwent PE between January 2016 (first PE data registered) and July 2023. The pediatric ICU is part of the IMIP, a high-complexity teaching hospital in Northeast Brazil that exclusively receives users of the public health system. The pediatric ICU includes 26 intensive care beds for clinical and surgical pediatric patients. There is no exclusive palliative care team, although the pediatric ICU team includes specialized pediatric palliative care professionals. This study did not include patients from the neonatal, oncology, or cardio-surgical ICUs.

Non probabilistic sampling was adopted, including for patients under 14 years of age who underwent PE in the pediatric ICU. Patients for whom PE was defined but who were accidentally extubated during handling by the medical team or by the patient themselves before the planned date were also included. Data collection was performed retrospectively using medical records.

The variables analyzed included demographic characteristics (sex and age), diagnosis at admission and the presence of chronic complex disease (CCD). A CCD is a medical condition that is expected to last at least 12 months and involves either several organ systems or 1 organ system requiring special pediatric care.^(11)^ Patients were also classified into five groups for palliative care on the basis of World Health Organization (WHO) definitions: Group 1, children with acute life-threatening conditions from which recovery may or may not be possible; Group 2, children with chronic life-threatening conditions that may be cured or controlled for a long period but that may also cause death; Group 3, children with progressive life-threatening conditions for which no curative treatment is available; Group 4, children with severe neurologic conditions that are not progressive but may cause deterioration and death; and Group 5, neonates who are severely premature or have severe congenital anomalies.

The following data were analyzed at PE indication: the clinical conditions associated with the indication, the length of hospital stay and IMV use, the use of invasive devices, and medications used. Severe neurological impairment was characterized by impaired motor and cognitive function, leading to dependence on caregivers and healthcare technologies for basic daily activities (e.g., feeding, communication, mobility, and maintenance of physiological functions).

The following variables related to the PE decision were analyzed: the time interval and number of family meetings between the PE indication and decision, and the associated LLSTs. After PE implementation, the following variables were analyzed: the time intervals between admission, PE indication, and PE implementation; the duration of IMV use; the professionals and family members present; the type of ventilation withdrawal; and the procedures performed before PE were analyzed. Finally, after PE, the use of respiratory support, symptoms and medication use, outcomes, and the interval between PE and death if it death occurred.

For the descriptive analyses, categorical variables are presented in tables as relative and absolute frequencies. Numerical variables are expressed as measures of central tendency and dispersion.

## RESULTS

Thirty-five patients underwent PE between January 2016 and July 2023 (seven years and six months), and eight medical records were unavailable. Thus, data from 27 patients were included in this study, and table 1 presents the patient characteristics. The patients were aged between five days and ten years (median of four months, interquartile range between two and 13 months), of which 51.8% were female.

**Table 1:** Characteristics of the patients who underwent palliative extubation

	Sex, Age	Pediatric ICU indication	CCD diagnosis	PPC WHO category	Previous PPC approach	PE indications	Symptoms after PE	Time to death after PE
01	Male,121 months	ARI, aspiration pneumonia	Adrenoleukodystrophy, epilepsy	3	No	Respiratory failure; Severe neurological impairment; Progression of underlying disease	-	5 days
02	Male,5 months	ARI, aspiration pneumonia	Microcephaly, CNS malformation	4	Yes	Respiratory failure, Severe neurological impairment	Dyspnea	7 days
03	Female,5 days	Postoperative gastroschisis, hypovolemic shock	-	5	Yes	Failure of curative therapeutic	-	3 days
04	Male,6 months	Septic chock	Down syndrome, PAH, CAVSD, ASD, VSD	2	Yes	Cardiocirculatory failure; Failure of curative therapeutics	-	8 days
05	Female,15 days	ARI, septic shock	Holoprosencephaly, Patau syndrome	5	No	Severe neurological impairment	-	0.7 hours
06	Female,17 months	CPA in anesthetic induction for elective cardiac catheterization	Down syndrome, PAH, CAVSD, ASD, VSD	2	Yes	Cardiocirculatory failure; Severe neurological impairment; Weaning failure from IMV	Dyspnea, agitation	9 days
07	Male,2 months	CPA in anesthetic induction for elective cardiac catheterization	Type IA pulmonary atresia	5	Yes	Cardiocirculatory failure; Intractable infection; Failure of curative therapeutic; Weaning failure from IMV	Dyspnea, agitation	3 days
08	Female,15 months	Septic shock, SARS	Joubert syndrome, epilepsy	4	Yes	Renal failure; Severe neurological impairment; Respiratory failure; Failure of curative therapeutic	Dyspnea, agitation, sialorrhea	-
09	Male,2 months	ARI, SARS, septic shock	NPCE, charge syndrome, ASD, PAH	4	Yes	Severe neurological impairment; Weaning failure from IMV	-	1 hour
10	Male,2 months	ARI	Jarcho-Levin syndrome	5	Yes	Respiratory failure; Severe neurological impairment; Weaning failure from IMV	-	3 hours
11	Male,1 month	Septic shock, enterocolitis, Postoperative intestinal perforation	-	1	Yes	Gastrointestinal failure; Severe neurological impairment; Failure of curative therapeutic;	-	12 days
12	Male,38 months	ARI, pneumonia	Niemann pick type C disease	3	Yes	Progression of underlying disease; Liver failure; Weaning failure from IMV	-	20 days
13	Male,11 months	Septic shock, pneumonia, HUS	-	1	Yes	Renal failure; Severe neurological impairment; Failure of curative therapeutics; Weaning failure from IMV	-	8 days
14	Female,2 months	ARI, congenital diaphragmatic hernia	-	5	Yes	Cardiocirculatory failure; Gastrointestinal failure; Respiratory failure; Failure of curative therapeutics; Weaning failure from IMV	-	0.3 hours
15	F,28 days	Postoperative period for gastroschisis, enterocolitis	-	5	No	Gastrointestinal failure; Failure of curative therapeutics	Agitation	28 days
16	Female,3 months	ARI, SARS	Bronchodysplasia, severe pulmonary stenosis	2	No	Respiratory failure, Severe neurological impairment; Weaning failure from IMV	Dyspnea, agitation	8 days
17	Female,3 months	Septic shock, pneumonia	Dextrocardia, RV hypoplasia, Single atrioventricular valve	5	Yes	Cardiocirculatory failure; Severe neurological impairment; LLST request by Family; Failure of curative therapeutic	-	3 hours
18	Male,2 months	SE, brain abscess, sagittal sinus thrombosis	-	1	Yes	Severe neurological impairment; Failure of curative therapeutic; Weaning failure from IMV	-	17 hours
19	Female,2 months	ARI	CNS Malformations, bilateral hydronephrosis	5	Yes	Respiratory failure; Severe neurological impairment; LLST request by family	-	1 hour
20	Male,5 months	SE, hypocalcemia	Epilepsy, multiple malformations, hypoparathyroidism	4	Yes	Respiratory failure; Severe neurological impairment; LLST request by family: Failure of curative therapeutics; Weaning failure from IMV	Dyspnea, agitation, seizure	4 days
21	Female,4 months	ARI, CPA	Corrected esophageal atresia, esophageal stenosis, ureteropelvic junction stenosis	5	Yes	Severe neurological impairment	Dyspnea	17 hours
22	Male,2 months	ARI, acute viral bronchiolitis	Osteogenesis imperfecta	3	Yes	Failure of curative therapeutics	Sialorrhea	-
23	Female,3 months	Decreased level of consciousness, meningitis, ventriculitis	Corrected myelomeningocele, hydrocephalus, Type II Chiari malformation	4	Yes	Respiratory failure; Severe neurological impairment; Failure of curative therapeutics; Weaning failure from IMV	Dyspnea	12 hours
24	Female,105 months	ARI, aspiration pneumonia, acute abdomen	Cerebral palsy	4	No	Respiratory failure; Malnutrition; Severe neurological impairment; Failure of curative therapeutic; Weaning failure from IMV	Dyspnea, agitation	24 hours
25	Female,103 months	ARI, septic shock, pneumonia	NPCE, West syndrome	4	No	Respiratory failure; Severe neurological impairment; Weaning failure from IMV	Dyspnea	38 days
26	Female,18 months	SE, decreased level of consciousness	Progressive epileptic encephalopathy	3	No	Severe neurological impairment; Progression of underlying disease; Failure of curative therapeutic	Dyspnea	3.6 hours
27	Male,9 months	Acute kidney failure	Kidney agenesis and dysplasia	2	Yes	Renal Failure; Intractable infection; Failure of curative therapeutics; Weaning failure from IMV	-	-

ICU - intensive care unit; CCD - complex chronic disease; PPC - pediatric palliative care; WHO–World Health Organization; PE - palliative extubation; ARI - acute respiratory insufficiency; CNS - central nervous system; PAH - pulmonary arterial hypertension; CAVSD - complete atrioventricular septal defect; ASD - atrial septal defect; VSD - ventricular septal defect; CPA - cardiopulmonary arrest; IMV - invasive mechanical ventilation; SARS - severe acute respiratory syndrome; NPCE - nonprogressive chronic encephalopathy; HUS - hemolytic-uremic syndrome; RV - right ventricle; LLST - limitation of life-sustaining therapies; SE - status epilepticus.

The most common reasons for admission to the pediatric ICU were acute respiratory failure (51.8%), septic shock (29.6%) and postoperative care (18.5%), and 85.1% of patients were admitted with MIV. Among the included patients, 77.8% had an underlying CCD, 44.4% had neurological impairment, and 22.2% had severe congenital heart disease. Six patients did not have chronic comorbidities at admission, three of whom were neonates admitted for surgical correction of severe noncardiac congenital malformations. All patients were classified into pediatric palliative care indication groups on the basis of their acute and chronic diagnoses at admission. In 74.1% of the cases, palliative care was discussed with the family before the indication for PE, and the topic was approached before admission to the pediatric ICU in 11.1% of the cases. In 25.9% of the cases, the first palliative care approach occurred concurrently with discussing PE with the family.

Table 2 describes the most frequent clinical conditions leading to PE indication, and the amount of time between admission to the pediatric ICU and PE indication, the definition and practice of PE and the time of MIV use are describe in table 3. The number of family meetings required to decide about PE ranged from one to four, with a decision being reached in a single meeting for 13 patients and within two meetings for eight patients.

**Table 2:** Conditions leading to the indication of palliative extubation

Condition	n (%)
Severe neurological impairment	19 (70.3)
Failure of curative therapeutic proposals	16 (59.2)
Weaning failure from IMV	14 (51.8)
Respiratory failure	11 (40.7)
Cardiocirculatory failure	5 (18.2)
Progression of underlying disease	3 (11.1)
Renal failure	3 (11.1)
Gastrointestinal failure	3 (11.1)
LLST request by family	3 (11.1)
Intractable/difficult to manage infection	2 (7.4)
Liver failure	1 (3.7)
Malnutrition	1 (3.7)

IMV - invasive mechanical ventilation; LLST - limitation of life-sustaining therapies.

**Table 3:** Characterization of the time intervals (in days) between admission to the pediatric intensive care unit, indication, implementation of palliative extubation, and in hospital death

	Min-Max	Median	IQR P25-75
Length of hospital stay and PE indication	1 - 66	14	5.5 - 32
Time between indication and first family conference	0 - 7	0	0 - 0,5
Time between the indication and definition of PE	0 - 36	2.5	0 - 7
Duration of IMV use at the time of PE indication	2 - 54	14	8 - 26
Duration of IMV use at the time of PE implementation	2 - 79	20	14 - 39
Time between PE and in hospital death	0 - 38	3	0 - 8

IQR - interquartile range; PE - palliative extubation; IMV - invasive mechanical ventilation.

All patients were on sedation or analgesic drugs at the time of PE indication, with fentanyl (51.8%) and midazolam (29.6%) being the most commonly prescribed drugs. The use of vasopressor drugs was observed in 14.9% of patients, and in 47.8% of patients, "do not initiate" was associated with PE. This was the most frequent LLST decision after a DNR order.

DNR orders were established with PE indication in 96.2% of the patients; this information was not found for one patient. LLSTs were determined for 70.3% of the patients, including 25.9% who decided to discontinue other life-sustaining therapies (e.g., vasopressor drugs, renal substitute therapies, antibiotics) beyond IMV. Documentation of limiting invasive procedures was found in 10 cases (37%).

The use of antibiotics at the PE indication was described in 16 patients, eight of whom discontinued the use of medication or decided not to change the medication regimen if failure or reinfection occurred. Infectious control was described as a criterion for defining the optimal time for PE in all patients except for two patients with defined untreatable infections.

Medical records indicated the presence of a physician for all PE procedures, a physiotherapist in 74.8% of procedures, a nursing technician in 66.6% of procedures and a nurse in 59.2% of procedures. The presence of a family member was reported in 70.3% of the cases (66.6% for mothers and 33.3% for fathers). In only one case, a seven-year-old sibling was authorized to be present for the procedure with their parents. The participation of other adult family members was allowed if the parents consented, but visits by children required an evaluation by psychology services.

Airway suction was described in 51.8% of the patients before PE, and sedative or analgesic administration was noted in 59.2% of the patients, dexamethasone in 48.1% of the patients and drugs for sialorrhea in 14.8% of the patients. The patient's diet was suspended in 29.6% of the patients.

The suspension of IMV was immediate (without a gradual reduction in parameters) in 51.8% of patients, and the modality was not identified in 37% of patients. In two patients, extubation occurred accidentally before the planned date, and noninvasive ventilation (NIV) was initiated until the family was informed, following planned palliative care with no recommendation for reintubation. All patients were using an endotracheal tube, which was removed concomitantly with IMV. No ventilatory support was used after PE in 51.8% of patients, oxygen therapy (nasal or facial mask) was used in 29.62% of patients, and NIV was used intermittently in 18.53% of patients.

After PE, 48.1% of patients presented symptoms such as dyspnea (84.6%), agitation (53.8%), and sialorrhea (11.1%). Death occurred in 88.8% of the children (74% in the pediatric ICU and 14.8% in the ward). The time to in-hospital death after PE ranged from 20 minutes to 38 days (Table 4). Three children (11.2%) were discharged from the hospital.

**Table 4:** Time to in hospital death after palliative extubation

Time	n (%)
≤ 1 hour	4 (16.6)
> 1 hour and ≤ 24 hours	7 (29.1)
> 24 hours and ≤ 5 days	4 (16.6)
> 5 days	9 (37.5)
Total in hospital deaths	24 (88.9)

## DISCUSSION

The present study describes a case series of 27 children and adolescents who underwent palliative extubation at a teaching hospital in the northeastern region of Brazil. Approximately 75% of the patients were aged 13 months or younger, and all of them were eligible for palliative care after pediatric ICU admission. The main condition related to the PE indication was severe neurological impairment, followed by therapeutic and IMV weaning failure. A family member was present during PE in 70.3% of the patients, 41.8% of the patients presented symptoms, and 88.8% progressed to death.

In the present study, although pediatric palliative care was indicated in all of the patients at admission, palliative care was only initiated in 11.1% of them before pediatric ICU admission, and in 25.9% of the cases, it occurred concurrently with discussing PE with the family. Multiprofessional teams still feel unprepared to indicate pediatric palliative care and LLSTs in Latin America. The palliative approach mostly occurs in the end-life stage, with DNR orders being the most common palliative care choice, whereas the suspension of life support, such as MIV, is rarely reported, even when death is the only possible outcome.^(12)^ In Brazil, the practice of LLSTs in end-of-life care has increased in recent decades, but reports of PE are scarce.^(13,14)^ A study conducted in southeast Brazil with healthcare professionals from three pediatric ICUs reported several barriers to performing PE, including the fear of legal processes, misinterpretation of PE as euthanasia, concerns about acceptance by families, and communication difficulties within the healthcare team.^(15)^

The present study revealed a high frequency of patients diagnosed with CCDs (77.8%) who experienced acute complications upon admission to the pediatric ICU, which is partially supported by the literature (50 to 100%).^(10,16,17)^ CCDs represent a large and heterogeneous group of life-threatening and life-limiting conditions, accounting for a significant portion of patient expenses and deaths in the pediatric ICU,^(18)^ which has motivated the discussion on pediatric palliative care and LLSTs.^(2)^ The indication for LLSTs is observed in cases of unfavorable progression of the underlying disease, even if it is an acute curable disease and despite all life support resources; conditions in which the prognosis is bad and there is no hope for cure or control; irreversible severe neurological sequelae; and, specifically, when IMV weaning has failed.^(10,16,17)^ Our findings corroborate the literature; in other words, PE is an indicator of severe and extensive disease, with an impact on functional and quality of life, a risk of early death, and high health support dependence, which are likely not modified by the maintenance of IMV.^(10,16-19)^

As observed in the present study, the literature reports PE indication for patients with or without CCDs, in acute unstable situations, in patients progressing to multiorgan compromise, and in patients where death is imminent and the continuation of IMV would prolong an irreversible process.^(10)^ This is often described for older adults and neonatal patients.^(4,20)^

Palliative extubation is also indicated for patients with chronic stable conditions who are dependent on IMV, as described in a Brazilian case series of 17 patients aged between five months and nine years with stable severe neurological and respiratory compromise. After the procedure, 57.9% of the patients died in the hospital within 15 minutes to five days after PE.^(17)^ In these cases, PE was indicated in contrast to maintaining IMV or tracheostomy to prolong life indefinitely, which does not affect quality of life and is correlated with a low probability of future weaning and withdrawal.^(19)^

The ability to maintain life-sustaining therapies for patients whose benefits are questionable can be harmful. The maintenance of IMV can burden healthcare systems^(21,22)^ and cause suffering for patients, families, and healthcare professionals.^(17,21)^ Additionally, IMV is not associated with better symptom control (e.g., dyspnea and cough) and requires painful procedures (e.g., aspirations, increased bed restriction), communication difficulties, regular monitoring, and technical care.^(21)^ Therefore, PE should be discussed as an alternative to management in ventilated patients as part of advanced palliative care plans.

In this study, in all patients, PE was a result of shared decision-making between the legal guardians of the patient and the healthcare team. PE decisions are often associated with other LLST definitions and the outcome of death. In Brazil, studies indicate that 32.8% to 66.1% of deaths in the pediatric ICU are associated with some form of LLST, with DNR orders being the most common.^(13,18,22,23)^

The number and duration of meetings and the involvement of patients and families in PE decision-making process remain heterogeneous in pediatric practice. Studies have reported active family involvement in 23% to 100% of cases.^(10,14,16,17,23-25)^ Sociocultural differences and the scarcity of documentation may explain this large variation. In contrast to Brazil, reports from England and the United States describe the family being present in all cases of PE, which is frequently performed in hospice facilities or in the patient's home out of the hospital.^(10,26)^ Furthermore, including the family in care planning, adopting clear and honest communication, and offering palliative care are associated with greater satisfaction and reduced conflicts and suffering in end-of-life care.^(7,26)^

This study reported that the length of hospital stay, duration of IMV use, and time interval between admission, indication, definition, and performance of PE varied, as reported in the literature.^(10,17)^ The indication for PE involves discussion with a multidisciplinary team, a rigorous review of clinical conditions and management possibilities, and discussion with the family and patient to understand their values and desires, thus aligning expectations and goals of care. After a decision, the team needs to check for any modifiable conditions beyond the main disease, such as treatable infections, that could lead to death or complications after PE; secure symptom control before, during and after the procedure; and provide the family and patients with time to fulfill their last wishes and prepare the team involved. The complexity of the decisions and actions involving PE and the individuality of each case justify the variability of the decision-making process duration.

The best moment to perform PE depends on optimizing comfort and symptom control and managing reversible conditions that could negatively influence the outcomes (e.g., infections, anemia, sedation). The team, family and patient should be prepared to address all possible outcomes, including prolonged survival and discharge to home. Time to perform religious or cultural rituals that are possible and desirable by the family and patient should also be a priority and goal of care.^(6-9,26,27)^ In this study, PE was preceded by a suspension of diet, airway suctioning, and the use of sedatives, analgesics, and dexamethasone; similar care practices have been reported and recommended elsewhere.^(7,9,27)^

The presence of symptoms after PE observed in this study is consistent with the literature, where dyspnea and agitation were the most commonly reported symptoms.^(10,17)^ Symptom control is one of the pillars of palliative care and should be a priority throughout the entire PE process.^(6)^ Although a reduction in ventilatory parameters before IMV withdrawal (terminal weaning) is associated with fewer symptoms,^(17,28)^ the present study emphasizes that sudden IMV withdrawal did not seem to increase symptom frequency.

In this study, 88.8% of patients died during hospitalization after PE, with a survival rate greater than five days in 37.5% of patients. Because PE removes life support and allows the disease to naturally progress, death is the most likely outcome, occurring in 57 - 100% of the cases,^(6,10,17,20)^ predominantly between minutes and five days after the procedure.^(6,10,16,17,20,25)^ Survival of less than one hour after the withdrawal of life support is associated with younger age (less than one month), the absence of spontaneous ventilation, the need for high ventilatory parameters, the use of high doses of vasoactive drugs, and the simultaneous withdrawal of multiple life support therapies.^(25,29,30)^ Predicting the time of death after PE could help families and healthcare teams better plan where and how to perform PE and care for patients.^(29,30)^

The present study has several limitations. First, a small sample size of patients who underwent PE as an LLST was studied. Although the procedure was coordinated by a pediatrician trained in palliative and intensive care, a pediatric palliative care team or specific PE protocol for pediatrics is unavailable at the Institution. In addition, as observed in other studies, data were collected exclusively from medical records, which are often not accurate.^(10,16)^ This study was conducted in a high-complexity teaching hospital linked to the public health system in the northeastern region of Brazil, which may not represent the reality of other services in the country. Nonetheless, this is the second Brazilian study on PE in children,^(17)^ and these findings may contribute to destigmatizing PE among the pediatric population, improving the clinical decision-making process and developing end-of-life palliative care plans.

Palliative extubation is an important procedure for patients on IMV with severe and irreversible conditions, as identified in this series of 27 cases, where all patients were eligible for palliative care upon admission to the pediatric ICU. Most of the patients were infants with CCDs, severe neurological impairments, inadequate responses or an absence of curative therapies or failure of IMV weaning. After PE, 48.1% of the patients presented symptoms, mainly dyspnea, agitation, and sialorrhea, all of which were controlled. Death in the hospital was the main outcome (88.8%), frequently occurring within five days of PE in 62.5% of the patients.

## CONCLUSION

Palliative extubation was mostly procedure performed in infants diagnosed with complex chronic conditions and severe and irreversible diseases, all indicated of whom were referred to other palliative care. Death in the hospital with controlling for some symptoms controlled was the main outcome.
